# Total hip replacement after gunshot hip injuries: a severity-based clinical framework—a single-centre experience

**DOI:** 10.1186/s13018-026-07037-2

**Published:** 2026-06-22

**Authors:** Mohamed Abo-Elsoud, Mostafa Ahmed Shawky, Mahmoud Fahmy

**Affiliations:** Pelvis Fracture and Arthroplasty Unit, Orthopaedic Department, Kasr Alainy Hospital, Cairo University, Cairo, Egypt

**Keywords:** Gunshot hip injuries, Total hip replacement, Reconstructive strategy, Staged arthroplasty

## Abstract

**Purpose:**

Gunshot hip injuries present major reconstructive challenges due to contamination, retained projectiles, soft-tissue compromise, and variable bone loss. Total hip replacement (THR) in this setting carries increased risks of infection, instability, and technical failure. This study evaluated the clinical and radiological outcomes of THR following gunshot hip trauma using a structured, severity-based subclassification to inform reconstructive strategy.

**Methods:**

Thirty patients with gunshot hip injuries underwent surgical debridement followed by THR, performed as single-stage or staged procedures according to injury severity, contamination, and systemic condition. Injuries were categorized into three groups: structurally benign injuries; severe or neglected injuries with major bone or soft-tissue compromise; and complex abdominopelvic traversing injuries with visceral involvement. Reconstruction predominantly used cementless acetabular components, with selective use of long femoral stems, revision cups, dual-mobility designs, and bone grafts or porous metal augments. Clinical outcomes were assessed using the Harris Hip Score (HHS) and WOMAC index, and radiological evaluation focused on component stability and graft incorporation.

**Results:**

A total of 30 patients were included: 11 in Group I (structurally benign injuries), 10 in Group II (severe or neglected injuries), and 9 in Group III (complex abdominopelvic traversing injuries). At a mean follow-up of 46 ± 10 (24–72) months, the mean Harris Hip Score (HHS) was 84 ± 10 and the mean WOMAC score was 19 ± 7. Operative complexity and blood loss increased with injury severity. Two-stage THR was required in 27% of Group I, 60% of Group II, and 100% of Group III. Radiographs demonstrated stable component fixation in all hips, with complete graft or augment incorporation within 6–12 months. Postoperative complications occurred in five patients, predominantly in higher-severity groups. Functional outcomes were inversely related to injury severity.

**Conclusions:**

THR after gunshot hip injury achieved satisfactory mid-term functional and radiological outcomes in this single-center cohort. Staged reconstruction and advanced implant strategies appear beneficial in managing complex and contaminated injuries. These findings are descriptive and intended to inform clinical decision-making; further studies are required for external validation.

## Introduction

Gunshot injuries to the hip pose a unique surgical challenge due to extensive bone loss, soft-tissue compromise, and contamination, which distinguish them from conventional osteoarthritis or post-traumatic degeneration [1, 2]. High-velocity projectiles commonly produce femoral head comminution, acetabular fractures, retained intra-articular fragments, devascularized bone, and devitalized musculature [3], increasing the risk of infection, mechanical instability, heterotopic ossification, and early prosthetic failure, while complicating both surgical planning and postoperative recovery [4, 5].

Despite the growing incidence of firearm-related trauma globally, evidence guiding total hip replacement (THR) in gunshot injuries remains limited. Most reports are small case series or single-case studies with heterogeneous populations, delayed presentations, and variable surgical techniques [6–8]. While staged THR, dual-mobility constructs, and modern bone grafting have been described, no consensus exists regarding patient selection, optimal timing, or standardized reconstruction algorithms [9, 10].

Understanding how injury severity, contamination, and soft-tissue compromise influence functional outcomes, complications, and implant survivorship is particularly important in conflict-affected or resource-limited regions, where initial management may be delayed or inadequate. A systematic, algorithmic approach can optimize recovery, minimize complications, and provide reproducible guidance for centers managing complex gunshot hip trauma.

Therefore, this study aimed to report clinical and radiological outcomes of THR following gunshot hip injuries and to describe a severity-based management framework derived from institutional experience, illustrating how injury patterns influenced surgical timing and reconstructive strategy.

### Patients and methods

This retrospective study included patients who underwent total hip replacement (THR) for gunshot hip injuries between January 2013 and December 2023. All patients were referred from surrounding war-zone regions for definitive management. Ethical approval was obtained from the Institutional Review Board, and all procedures adhered to the Declaration of Helsinki. Written informed consent for surgical treatment, imaging, and use of anonymized clinical data for research and publication was obtained from all patients at the time of treatment.

**Inclusion criteria:** Patients aged ≥ 16 years with gunshot injuries involving the hip joint who subsequently developed post-traumatic arthritis, avascular necrosis, or non-reconstructable femoral head or acetabular damage requiring total hip replacement. All included patients were deemed unsuitable for joint-preserving procedures at the time of presentation.

**Exclusion criteria**: Patients with hip arthritis of non-traumatic etiology, incomplete clinical or radiological records, or follow-up shorter than 24 months.

### Preoperative preparation

All patients underwent standard radiographic assessments including anteroposterior pelvis and lateral hip radiographs. CT scans were used to define acetabular and femoral bone loss, fragment location, articular destruction, and projectile trajectory. MRI was reserved for patients with suspected abdominal or pelvic contamination, especially those with missile trajectories passing through the bowel, bladder, or perineum, and to assess the extent of infection in some cases. Multidisciplinary consultations with general and colorectal surgery, urology, vascular surgery, and infectious disease were performed according to associated injuries and patient clinical status. Laboratory evaluation included inflammatory markers, cultures where indicated, and infection workup in patients with chronic wounds or persistent drainage.

### Framework development and injury pattern definitions

The cohort demonstrated marked heterogeneity in entry site, projectile trajectory, missile type, degree of bone and soft-tissue injury, retained foreign bodies, and associated visceral or pelvic organ involvement. Injury patterns were classified using objective clinical and radiological parameters routinely assessed during management, including: (1) extent of acetabular and femoral bone loss on CT, (2) integrity of the soft-tissue envelope, (3) presence of visceral or pelvic organ injury, and (4) evidence of contamination or infection.

As no validated classification system exists for gunshot-related hip injuries, patients were descriptively grouped based on recurring combinations of these objective features observed during clinical care. The severity-based subclassification was developed retrospectively and reflects the reconstructive strategies actually employed in response to injury severity, contamination, and associated visceral involvement. Importantly, this framework was not used prospectively or as a predictive decision-making tool. Allocation to single-stage versus staged THR, implant selection, and use of revision constructs was guided by predefined clinical criteria including contamination status, visceral injury, soft-tissue integrity, and bone loss. The subclassification was applied retrospectively for descriptive and comparative analysis only.

Patients were categorized into three clinical groups according to injury severity and reconstructive complexity:

### Group I—Structurally benign gunshot hip injuries

**Fig. 1:**
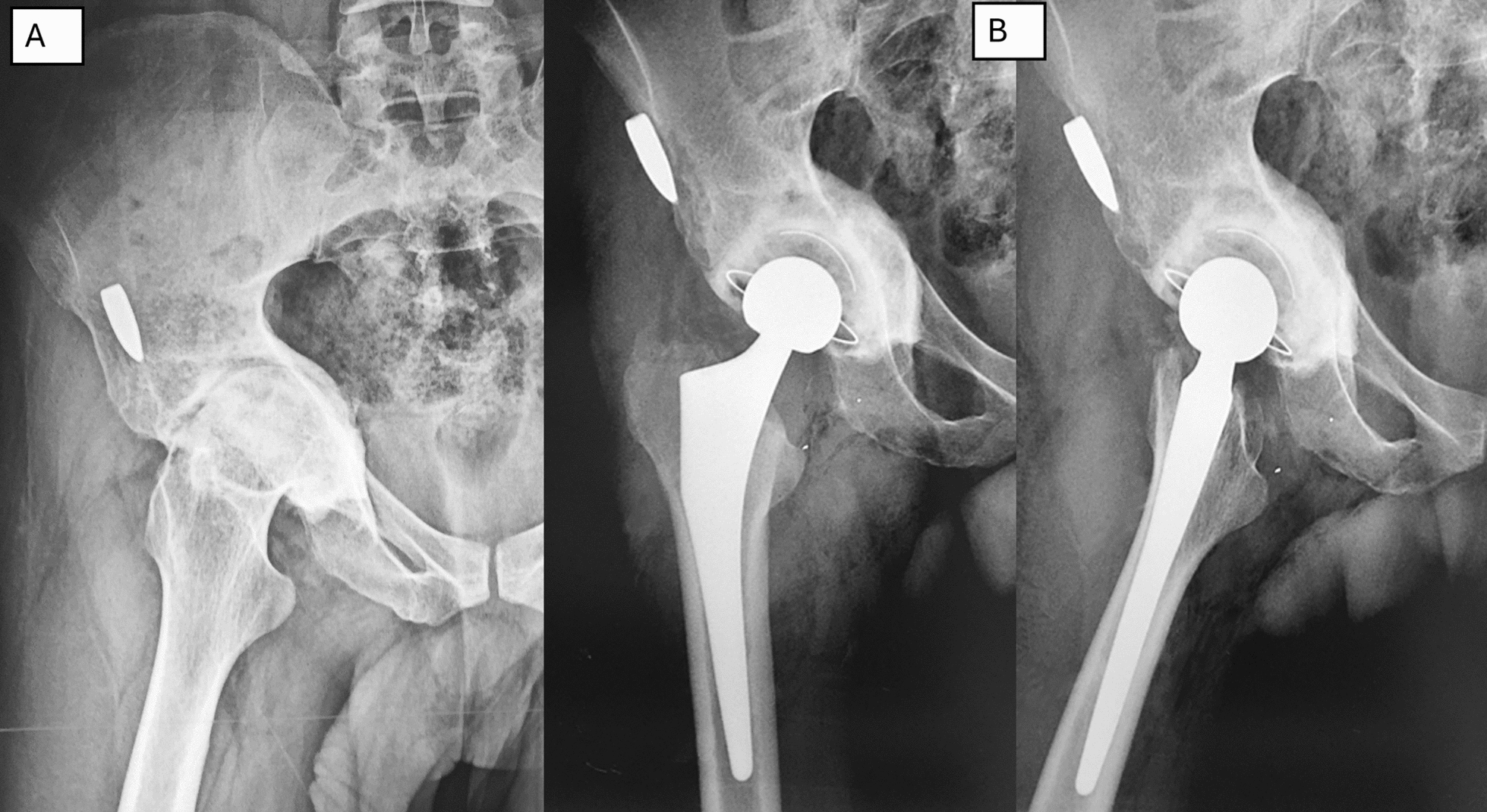
A 24-year-old patient who sustained a gunshot injury traversing the pelvis (Group I), with no exit wound and a retained projectile in the iliac bone, presenting 11 months after injury. **A** Preoperative AP pelvic radiograph demonstrating retained bullet within the iliac bone with secondary degenerative changes of the hip joint. **B** Final follow-up radiograph at 25 months following single-stage debridement and cementless total hip replacement demonstrating satisfactory implant positioning, Stable component fixation with no evidence of loosening

Acute injuries with minimal bone loss, intact soft tissues, and no visceral or pelvic contamination. The acetabulum and proximal femur were largely preserved, allowing straightforward reconstruction with prompt debridement, removal of retained fragments, and typically single-stage THR (Fig. 1 case example).

### Group II—Severe and neglected gunshot hip injuries

**Fig. 2:**
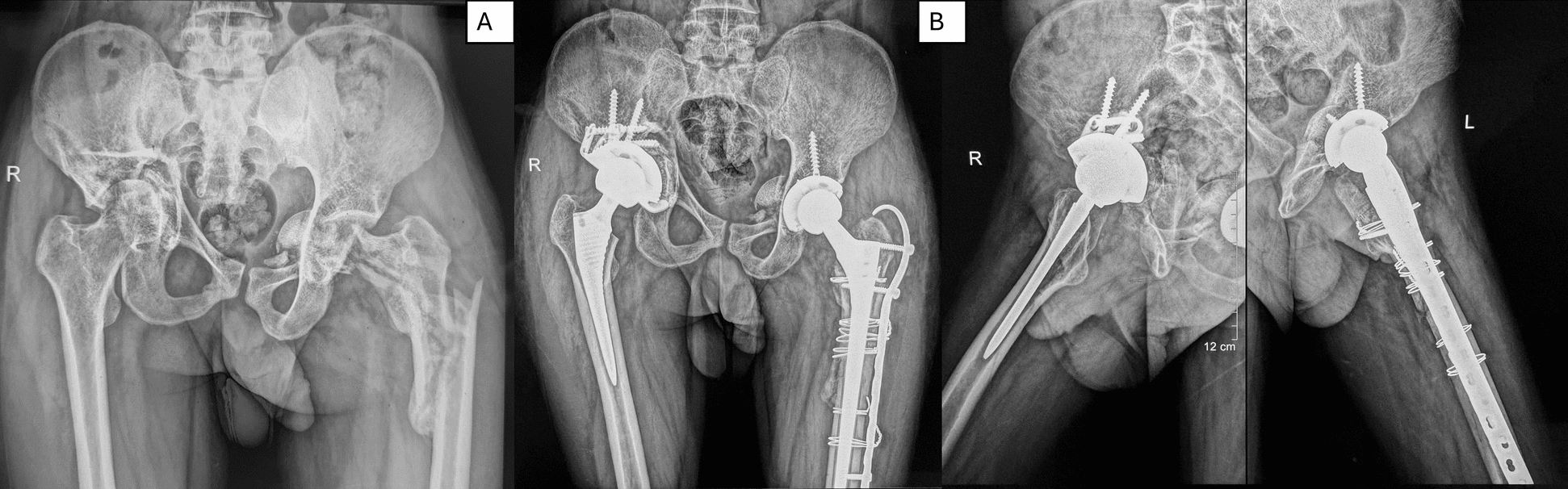
A 32-year-old patient who sustained a gunshot injury traversing the pelvis without abdomino-pelvic visceral injury presented after 1 year of injury—complex neglected injury **(Group II). A** Preoperative anteroposterior pelvic radiograph demonstrating a neglected right acetabular fracture with femoral head erosion, and a neglected left femoral head–neck fracture associated with proximal femoral fracture. **B** Final follow-up radiograph **at 30 month** showing bilateral total hip arthroplasty, with a revision trabecular titanium acetabular cup on the right side and a cementless long femoral stem with trochanteric plate fixation on the left side. Injury 1 year earlier to THR

Extensive bone loss, severe soft-tissue compromise, or delayed presentation. Patterns included large acetabular defects, pelvic discontinuity, proximal femoral destruction, chronic dislocation, ankylosis, and limb shortening. Reconstruction often required porous metal augments, femoral head autografts, revision cups, long stems, and dual-mobility components. Some cases required soft tissue releases and subtrochanteric femoral shortening osteotomy to restore length and enable safe reduction (Fig. 2 case example).

### Group III—Complex abdominal–pelvic traversing injuries

**Fig. 3:**
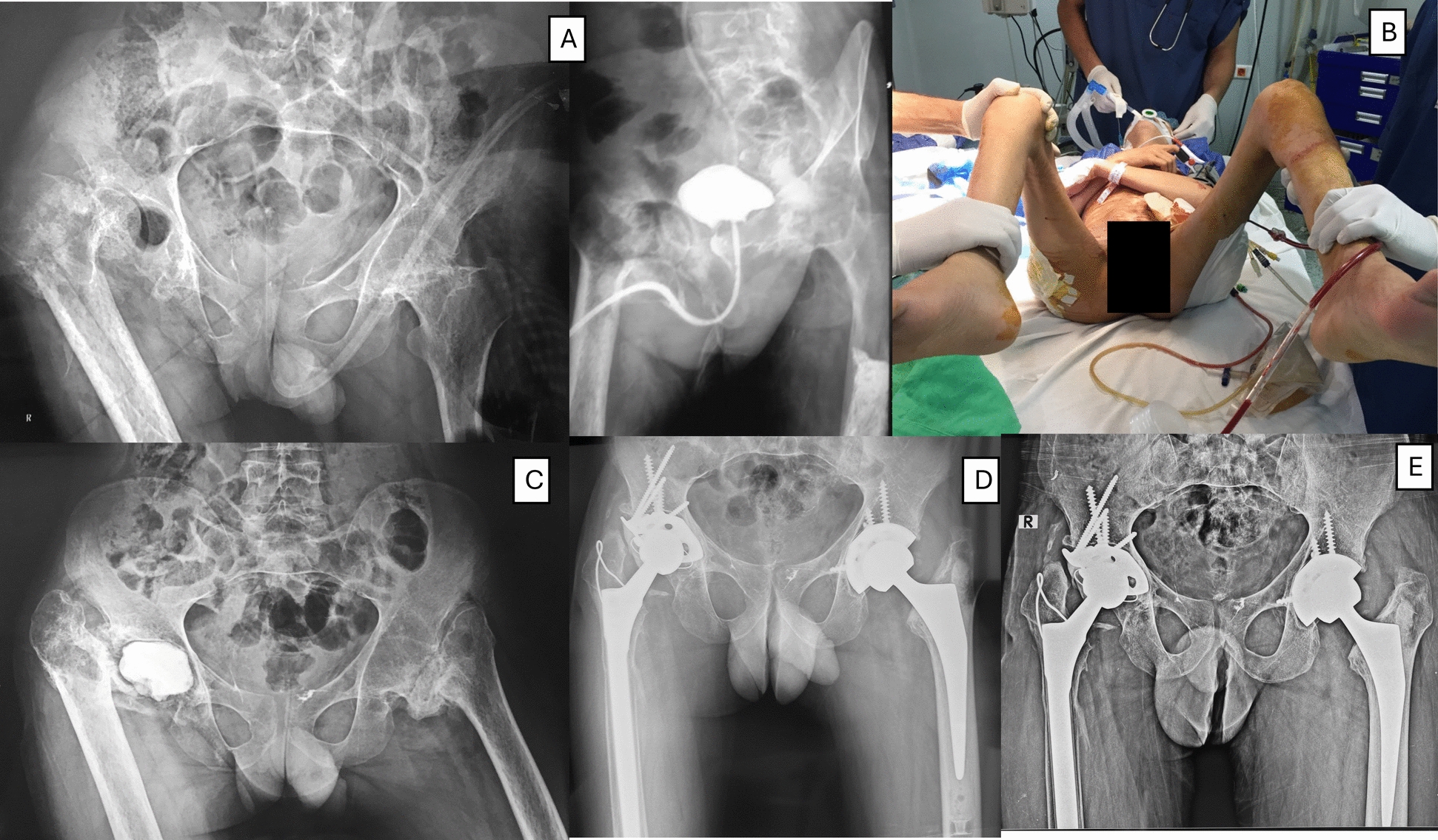
A 18-year-old male who sustained a gunshot injury traversing the abdomen **(Group III),** resulting in a right hip fracture, and presented with delayed referral. **A** Preoperative radiograph demonstrating a neglected right femoral neck fracture and septic hip and a contralateral fused hip. **B** Preoperative clinical photograph showing fixed deformity of both hips. **C** Immediate postoperative radiograph following the first-stage procedure, illustrating right hip debridement and an in situ left femoral neck osteotomy performed to facilitate subsequent urological reconstruction.** D** Final radiograph at 4-year follow-up demonstrating stable bilateral total hip arthroplasty

**Fig. 4:**
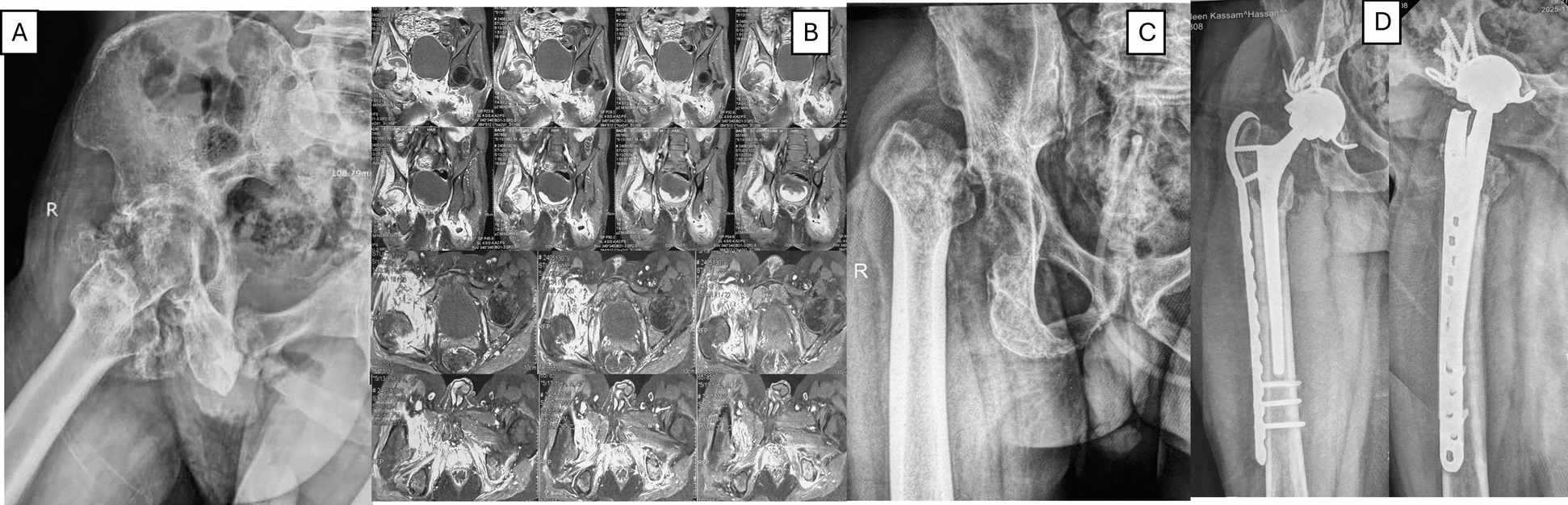
A 28-year-old patient who sustained a gunshot injury traversing the abdomen **(Group III),** resulting in a right hip injury with delayed referral, associated with perineal wound and colonic injuries. **A** Preoperative radiograph demonstrating right hip destruction with acetabular erosion. **B** Preoperative MRI showing associated uro-colonic injury with pelvic abscess formation and post-septic osteolysis of the right hip. **C** Postoperative radiograph following extensive debridement of the right hip, bilateral ischial tuberosities, and perineal wound, performed in collaboration with the colorectal surgery team, who carried out abdominal debridement, colostomy creation, and perianal/perineal wound debridement. **D** Final follow-up at 40 months from injury (32 months after THR) demonstrating stable implant positioning. Injury 8 months earlier to THR

Projectiles traversed the abdomen before the pelvis, causing combined visceral and musculoskeletal trauma (e.g., colonic/rectal perforation, perineal contamination, bladder or urethral injury). Preoperative MRI assessed for colo-articular fistula. Management was staged: aggressive debridement, foreign-body removal, and coordinated colorectal or urologic interventions (often colostomy), followed by delayed THR after infection control and systemic stabilization; arthroplasty was postponed until stoma closure when present (Figs. 3 and 4 case examples).

**Surgical technique:** All procedures were performed by senior surgeons specialized in complex arthroplasty and pelvic trauma. Patients were positioned in the lateral decubitus position under general or spinal anesthesia, using a posterolateral approach.

In patients requiring staged reconstruction, the initial procedure focused on thorough debridement and infection control. This included extensive excision of all devitalized soft tissue and necrotic bone, meticulous removal of intra-articular and extra-articular foreign bodies (including bullet and shell fragments), and evacuation of hematoma or purulent collections when present. Particular attention was paid to the hip joint capsule, surrounding musculature, and potential missile tracts. Copious irrigation was performed using large volumes of normal saline, with or without antiseptic solutions according to contamination severity. In cases with suspected or confirmed infection, multiple deep tissue samples were obtained for microbiological analysis.

In patients with abdominopelvic traversing injuries, debridement was performed in coordination with general or colorectal surgeons, addressing associated visceral injuries, controlling contamination, and, when indicated, performing diversion procedures such as colostomy.Temporary measures included delayed wound closure or use of negative pressure wound therapy when soft-tissue conditions were suboptimal. Definitive total hip replacement was deferred until adequate infection control, soft-tissue recovery, and systemic stabilization were achieved.

Core principles applied in all cases included: meticulous removal of intra-articular bullets, shell fragments, and necrotic tissue; extensive irrigation to minimize contamination; preference for cementless acetabular components; use of acetabular reconstruction and revision cups in cases with segmental defects, pelvic discontinuity, or deficient bone stock; use of porous metal augments or impaction grafting for acetabular defects; femoral stem selection based on canal morphology and bone stock; explicit criteria guided the selection of dual-mobility components, revision cups, and stem length depending on bone loss, soft-tissue status, and risk of instability; tailored antibiotic prophylaxis based on contamination level.

Single- versus two-stage THR was guided by injury severity, contamination, visceral involvement, and systemic condition. Two-stage THR was used for infection, gross contamination, bowel or perineal involvement, with initial debridement and delayed THR. Single-stage THR was performed when contamination or infection was absent, combining debridement with immediate implantation [Table 1].

**Table 1:** Injury severity group and corresponding reconstructive strategy

Group	Key features	Timing	Strategy
I	No contamination, minimal bone loss	Single stage	Immediate THR
II	Bone loss, soft-tissue compromise	Selective staging	Revision THR
III	Visceral contamination	Mandatory staging	Delayed THR

### Postoperative management

Postoperative Management, including early mobilization was encouraged, with weight-bearing advanced according to bone quality, fixation stability, and the extent of reconstruction. Antibiotics were continued when bowel contamination or soft-tissue infection was present, guided by infectious disease specialists. Wound care and drain management were tailored according to tissue integrity and contamination risk.

### Radiographic evaluation

Standardized radiographic assessment included anteroposterior (AP) pelvis and lateral hip views at each follow-up interval. Radiographs were independently reviewed by two orthopaedic surgeons who were not involved in the surgical procedures. In cases of disagreement, consensus was reached through joint evaluation.

Radiological evaluation was performed immediately postoperatively, at 6 weeks, 3 months, 6 months, 12 months, and annually. Assessment included: component position and alignment, osseointegration based on the Engh criteria, graft incorporation, presence of radiolucent lines or migration, heterotopic ossification classified using the Brooker grading system, evidence of loosening or mechanical failure.

### Functional outcome assessment

Functional outcomes were assessed using the Harris Hip Score (HHS) and the WOMAC index. The HHS evaluates pain, function, deformity, and range of motion, with a maximum score of 100 points, where higher scores indicate better function. The WOMAC index assesses pain, stiffness, and physical function, with lower scores indicating better clinical outcomes. Patient satisfaction was assessed using a subjective three-tier scale (satisfied, partially satisfied, unsatisfied). Mobility status was evaluated based on ambulatory capacity and categorized as independent ambulation, ambulation with assistive devices, or non-ambulatory. Clinical assessments were performed by two independent orthopaedic surgeons not involved in the index procedures to minimize observer bias. All complications were systematically recorded.

Postoperative outcomes were independently assessed by two orthopaedic surgeons who were not involved in the surgical treatment or postoperative care of the patients. Inter-observer reliability was evaluated using the intraclass correlation coefficient (ICC) for continuous variables and weighted Cohen’s kappa for categorical variables. Agreement was excellent for both functional outcome measures and ambulatory status, with an ICC of 0.94 (95% CI 0.88–0.97) for the Harris Hip Score, an ICC of 0.92 (95% CI 0.85–0.96) for the WOMAC score, and a weighted kappa coefficient of 0.89 (95% CI 0.77–1.00) for ambulatory status classification.

### Statistical analysis

SPSS v26 was used. Continuous data are mean ± SD and tested for normality (Shapiro–Wilk). Group comparisons: ANOVA for continuous, chi-square/Fisher’s exact for categorical. Correlations between injury severity and HHS/WOMAC were assessed with Spearman’s ρ. Significance:* p* < 0.05, two-tailed. Inter-observer reliability was assessed using intraclass correlation coefficients (ICC) with 95% confidence intervals for continuous variables and weighted Cohen’s kappa coefficients with 95% confidence intervals for categorical variables.

## Results

**Fig. 5:**
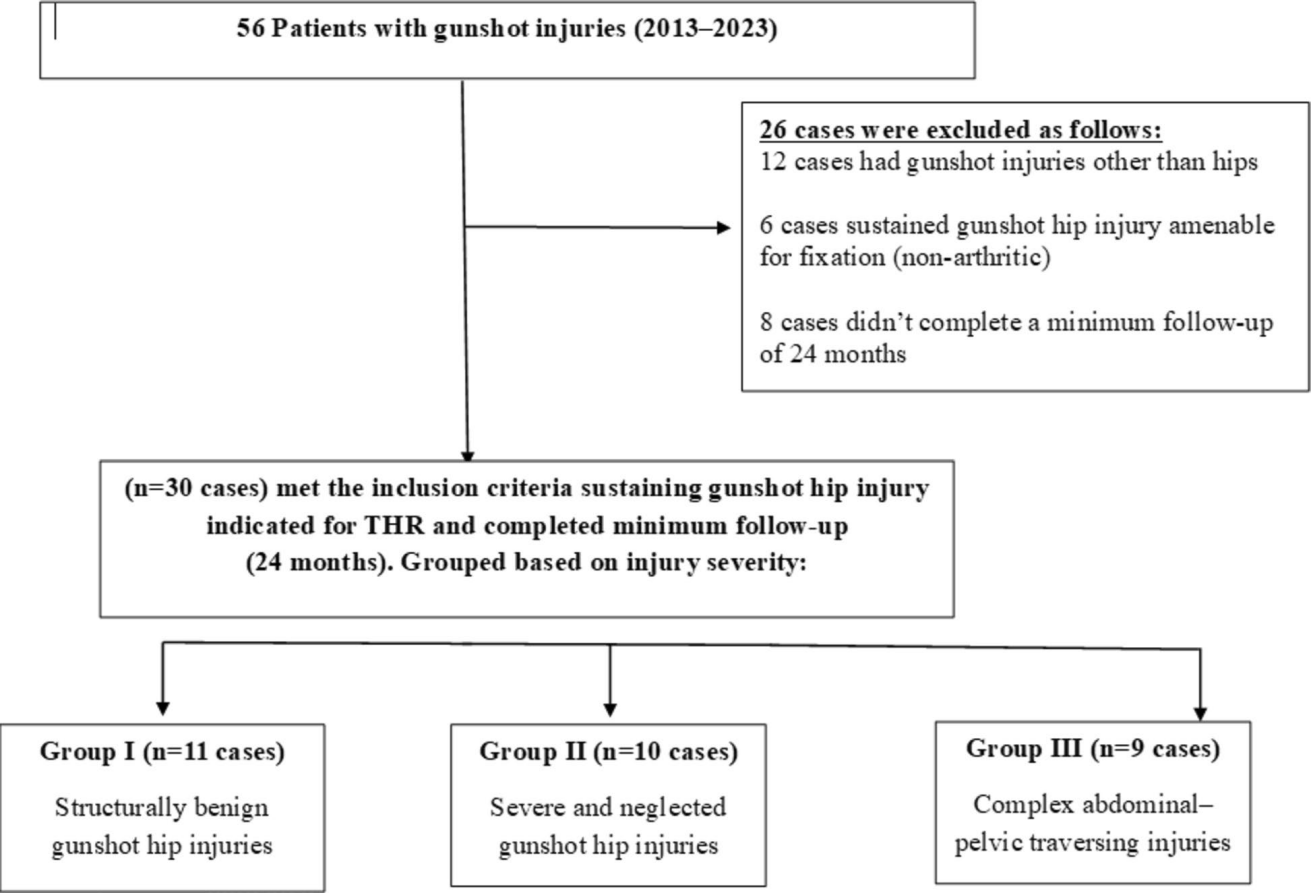
Study flow diagram of patient selection

A total of 30 patients (22 males, 8 females) underwent total hip replacement (THR) following gunshot hip injuries**.** All patients were referred from nearby conflict-affected regions and initially underwent surgical debridement for contamination, retained fragments, or soft-tissue compromise as indicated by injury characteristics (Fig. 5). THR was performed either as a single-stage or staged procedure depending on injury severity, presence of contamination, and systemic status. The mean age at surgery was 36 ± 11 years (range, 18–62 years), and the mean follow-up period for this cohort was 46 ± 10 months (range, 24–72 months). The mean follow-up duration differed slightly among the injury groups, reflecting variations in referral patterns and treatment complexity. Mean follow-up was 43 ± 8 months in Group I, 46 ± 9 months in Group II, and 50 ± 11 months in Group III.

Three patients underwent bilateral THR, performed sequentially.

The mean operative time was 171 ± 34 min, and the mean intraoperative blood loss was 686 ± 244. Cementless acetabular components were used in most of the patients. Eleven patients required additional acetabular reconstruction using porous metal augments (n = 5) or bone grafts (n = 6). Long-stem femoral implants were utilized in 14 patients, predominantly in cases with severe bone loss or compromised soft tissues. Revision cups were used in 6 patients.

Dual mobility acetabular cups were employed in 7 patients, mainly in group 2 and 3, to mitigate instability risk.

Based on injury severity and reconstructive complexity, patients were categorized into three groups. Two-stage THR was performed in 3 of 11 patients (27%) with structurally benign injuries (Group I), 6 of 10 patients (60%) with severe and neglected injuries (Group II), and all patients (9/9, 100%) with complex abdominopelvic traversing injuries (Group III). The mean operative time and blood loss increased progressively with injury severity. At the final stage of THR implantation, operative times were 138 ± 20 min, 188 ± 26 min, and 188 ± 28 min, and corresponding blood loss of 450 ± 120 ml, 750 ± 180 ml, and 900 ± 170 ml in Groups I, II, and III, respectively. Injury severity was directly associated with surgical timing and reconstructive complexity. Single-stage THR was predominantly performed in Group I, whereas staged reconstruction was increasingly required in Groups II and III. Use of revision components, long femoral stems, and dual-mobility cups increased with injury severity, reflecting greater bone loss and soft-tissue compromise.

At final follow-up, patients demonstrated satisfactory functional improvement. The mean Harris Hip Score (HHS) for the entire cohort was 84 ± 10, and the mean WOMAC score was 19 ± 7. Functional outcomes showed a clear inverse relationship with injury severity. Patients with structurally benign injuries achieved the best results (HHS 92 ± 5, WOMAC 12 ± 3), followed by the severe and neglected group (HHS 81 ± 7, WOMAC 21 ± 5), while patients with complex abdominopelvic injuries demonstrated comparatively lower but still satisfactory outcomes (HHS 78 ± 8, WOMAC 24 ± 6).

Radiographically, all hips demonstrated stable component fixation at final follow-up. All porous metal augments and structural grafts showed radiographic incorporation within 6–12 months. Heterotopic ossification occurred in four patients (13%), predominantly Brooker grade I–II.

Postoperative complications occurred in five patients. These included two superficial wound infections, three deep infections requiring surgical debridement with implant retention. Complication rates were lowest in the structurally benign group and increased with injury severity. No perioperative mortality, neurovascular injury, or implant failure was recorded.

A comparative summary of operative parameters, reconstruction strategies, functional outcomes, and complications across the three injury groups is presented in Table 2.

**Table 2:** Summary of operative parameters, implant use, functional outcomes, and complications across gunshot injury groups

Variable	Group I—Structurally benign injuries (n = 11)	Group II—Severe & neglected injuries (n = 10)	Group III—Complex abdominopelvic injuries (n = 9)	Total (N = 30)
Two-stage THR	3/11 (27%)	6/10 (60%)	9/9 (100%)	18/30 (60%)
Operative time (min)	138 ± 20	188 ± 26	188 ± 28	171 ± 34
Blood loss (mL)	450 ± 120	750 ± 180	900 ± 170	686 ± 244
Dual-mobility cup	0 (0%)	4 (40%)	3 (33%)	7 (23%)
Long-stem femoral implant	0 (0%)	7 (70%)	7 (78%)	14 (47%)
Revision cups	0 (0%)	2 (20%)	4 (44%)	6 (20%)
Porous metal augment / bone graft	0 (0%)	7 (70%)	4 (44%)	11 (37%)
Shortening osteotomy	0 (0%)	5 (50%)	0 (0%)	5 (17%)
HHS (final follow-up)	92 ± 5	81 ± 7	78 ± 8	84 ± 10
WOMAC (final)	12 ± 3	21 ± 5	24 ± 6	19 ± 7
Heterotopic ossification	1 (9%)	2 (20%)	1 (11%)	4 (13%)
Complications				
Superficial infection	0 (0%)	1 (10%)	1 (11%)	2 (7%)
Deep infection	0 (0%)	2 (20%)	1 (11%)	3 (10%)
Radiographic stability	11/11 (100%)	10/10 (100%)	9/9 (100%)	30/30 (100%)
Graft/Augment incorporation (6–12 months)	–	7/7 (100%)	4/4 (100%)	11/11 (100%)
Mean follow up	43 ± 8	46 ± 9	50 ± 11	46 ± 10 months

## Discussion

Total hip replacement (THR) after gunshot trauma is technically challenging due to bone destruction, soft-tissue compromise, and contamination. High-velocity projectiles often cause femoral head comminution, acetabular fractures, retained fragments, and devascularized bone and muscle, increasing risks of infection, instability, heterotopic ossification, and early implant failure [1–4]. The decision between acute and staged arthroplasty depends on injury severity, contamination, and visceral involvement, with staged procedures generally required for bowel injury, extensive soft-tissue necrosis, or intra-articular contamination [3, 4]. Essential prerequisites for THR include thorough debridement, infection control, fragment removal, and accurate bone loss assessment [5–7]. Modern reconstructive techniques—such as porous metal augments, impaction grafting, long-stem components, and dual-mobility bearings—help restore bone stock and implant stability, though complication rates remain higher than conventional arthroplasty [5–12].

This study represents one of the largest single-center experiences of THR following gunshot hip injuries from a conflict-affected region. Located within an active war zone, our institution functioned as a major referral center for patients from surrounding areas, many of whom presented after delayed or inadequate initial management due to disrupted healthcare systems. Clinical decision-making was guided exclusively by patient safety and standard-of-care principles, independent of research considerations. Injury patterns were highly heterogeneous, ranging from isolated fractures to extensive pelvic and proximal femoral destruction, frequently associated with intra-abdominal or genitourinary injuries. This variability, reflecting differences in projectile trajectory, contamination, retained fragments, and prior interventions, complicates standardized management [8].

Currently, no validated classification system exists for gunshot-related hip injuries requiring arthroplasty. Traditional fracture and acetabular classifications do not adequately account for contamination, projectile trajectory, or visceral involvement—factors that critically influence reconstructive strategy in this context. Accordingly, the framework presented here was developed to complement, rather than replace, established classifications by incorporating soft-tissue integrity and contamination variables relevant to arthroplasty planning.

Based on recurring patterns observed in our cohort, patients were grouped into: Group I—structurally benign injuries amenable to single-stage THR; Group II—severe or neglected injuries requiring complex reconstruction and, in selected cases, staged procedures; and Group III—complex abdominopelvic traversing injuries necessitating staged THR with coordinated abdominal or urologic management. This framework emphasized key diagnostic considerations, particularly the role of MRI in excluding occult visceral involvement or persistent contamination, and supported a stepwise approach to imaging, surgical timing, implant selection, and soft-tissue management. While this structured approach proved useful within our institutional experience, it was applied descriptively and retrospectively. Its reproducibility and effect on clinical outcomes were not formally evaluated, and further studies are required to determine whether such frameworks improve outcomes compared with alternative strategies.

Although Group I injuries were described as structurally benign, it is important to emphasize that all patients included in this study presented with established post-traumatic sequelae at the time of definitive management, including advanced cartilage damage, femoral head collapse, avascular necrosis, or secondary arthritis. Therefore, joint-preserving procedures such as internal fixation were not considered appropriate in this cohort. In acute settings with preserved articular surfaces and minimal contamination, internal fixation may represent a viable treatment option. However, gunshot injuries frequently involve occult chondral damage, intra-articular contamination, and compromised vascularity, which increase the risk of fixation failure and subsequent degenerative changes. In addition, many patients in this series were referred after delayed or inadequate initial management, further limiting the feasibility of reconstructive fixation. Consequently, total hip replacement was considered the most reliable option to restore function and alleviate pain in this patient population.

The occurrence of infectious complications predominantly in patients with complex abdominopelvic injuries highlights the critical role of contamination and soft-tissue compromise in determining outcomes. While our current protocol emphasizes meticulous debridement, staged reconstruction, and targeted systemic antibiotic therapy, the potential role of adjunctive local antimicrobial strategies warrants consideration. Techniques such as antibiotic-loaded bone cement or local antibiotic carriers (e.g., calcium sulfate-based delivery systems) may provide additional infection control in selected high-risk cases. Similarly, specialized irrigation and decontamination solutions have been proposed to reduce bacterial burden. However, the routine use of these adjuncts remains controversial due to limited high-level evidence and variability in clinical practice. Based on our findings, we have not adopted these measures as standard practice but consider them selectively in cases with severe contamination or borderline infection control. Further prospective studies are required to define their role in the management of gunshot-related hip injuries.

When compared with the work of Pazarci et al. [2], important conceptual differences emerge that highlight the added value of our proposed subclassification. Pazarci et al. reported a small series of patients undergoing delayed total hip arthroplasty after gunshot injury to the hip joint, emphasizing soft-tissue recovery and reporting acceptable functional outcomes. However, their study primarily focused on the timing of arthroplasty and postoperative results, without providing a structured framework for preoperative risk stratification, injury severity assessment, or decision-making regarding staged versus single-stage reconstruction. In contrast, our classification is injury-pattern driven rather than timing-based, integrating projectile trajectory, degree of osseous destruction, soft-tissue compromise, contamination, and—critically—abdominopelvic visceral involvement.

A key advantage of our approach is the systematic incorporation of advanced imaging, particularly the mandatory use of MRI in suspected abdominopelvic traversing injuries (Group III), enabling detection of occult bowel or urogenital involvement that may be underestimated on CT alone. This distinction has direct clinical implications, as unrecognized visceral injury substantially alters infection risk, staging strategy, and timing of reconstruction. Furthermore, by stratifying patients into three reproducible groups with clearly defined management pathways, our subclassification allows predictable selection of surgical timing, implant strategy, and need for staged reconstruction, rather than reliance on individual surgeon judgment alone.

While Pazarci et al. demonstrated that delayed THR can yield satisfactory outcomes in selected cases, our study extends this concept by offering a comprehensive, algorithmic framework applicable across the full spectrum of gunshot hip injuries—from structurally benign fractures to complex abdominopelvic traversing trauma. This structured approach enhances reproducibility, improves patient selection, and may reduce avoidable complications, particularly in conflict zones and resource-limited trauma settings.

In our 30 patients, functional recovery was achieved across all injury severities, with mean HHS 84 ± 10 and WOMAC 19 ± 7 (Groups I–III: HHS 92 → 81 → 78; WOMAC 12 → 21 → 24). Surgical complexity, operative time, blood loss, and complication rates increased with injury severity, higher in Groups II–III. Implant selection matched injury patterns, and all implants and grafts incorporated radiographically within 6–12 months. Overall complications occurred in 16.7%. Functional gains were greatest in structurally benign injuries and progressively lower, yet clinically meaningful, in complex cases, highlighting the effectiveness of staged and tailored reconstructive strategies even in severely damaged hips. The success of staged THR in this cohort underscores the critical importance of meticulous initial debridement and infection control as a prerequisite for definitive reconstruction.

Abialevich et al. [1] reported 12 patients undergoing staged THR after severely contaminated gunshot wounds, using antibiotic spacers to control infection; 2 patients developed superficial wound infections, but functional outcomes were favorable. Pazarci et al. [2] described 8 patients who underwent delayed THR after soft-tissue healing, with one case of early postoperative infection. Özden et al. [3] analyzed 26 patients injured in the Syrian civil war, noting significant functional improvements, although infection occurred in 4 cases, primarily in shell-fragment injuries.

Maqungo et al. [5] performed surgical hip dislocation to remove retained intra-articular bullets in 6 patients, reporting accelerated cartilage degeneration if fragments were left in situ but no major infections. Naziri et al. [6] studied 9–12 patients undergoing primary THR for post-traumatic arthritis, noting chronic synovitis from retained fragments and one case of prosthetic loosening.

Older series provide context for evolving practices. Sclafani and Lachiewicz [11] reported 5 cases of lead-induced arthropathy, emphasizing fragment-related cartilage damage. Long et al. [10] and Sethi et al. [7] collectively reviewed 20–25 patients, highlighting delayed THR after soft-tissue recovery as critical to optimizing outcomes. Najibi et al. [8] detailed 16 acetabular gunshot injuries, illustrating technical challenges and perioperative complications including infection and heterotopic ossification. Bell et al. [12] reported a case of a femoral neck fracture caused by a gunshot wound treated with a staged total hip arthroplasty approach, demonstrating successful infection control and satisfactory functional recovery, thereby supporting staged reconstruction in complex or contaminated gunshot-related hip injuries.

The functional outcomes observed in this study are consistent with those reported in previously published series of total hip arthroplasty following gunshot-related hip injuries. In the present cohort, the mean Harris Hip Score (HHS) was 84 ± 10, which is comparable to values reported by Pazarci et al. [2] and Özden et al. [3], who demonstrated satisfactory postoperative functional recovery despite varying injury severity and treatment strategies. Similarly, Abialevich et al. [1] reported favorable functional outcomes following staged arthroplasty in contaminated injuries, supporting the effectiveness of this approach. [Table 3].

**Table 3:** Comparative outcomes of Total Hip Replacement (THR) after gunshot hip injuries

Study (Ref)	N (THR cases)	Follow-up (mo)	Complications	Functional outcomes	Key findings
Present study	30	46 ± 10 (24–72)	5/30 (16.7%); superficial infection 2/30: 6.7% deep infection 3/30 (10%)	At final follow-up: HHS: 84 ± 10; WOMAC: 19 ± 7	Severity-based framework enabled tailored single vs staged THR with 100% radiographic stability; outcomes inversely related to injury severity; acceptable mid-term results despite complex injury patterns
Abialevich et al*.* [1]	2	~ 12–24	None major	Full functional recovery described	Two staged THA after severe gunshot trauma yielded excellent recovery
Pazarci et al*.* [2]	10	Retrospective (exact not stated)	Infection: 1/10	HHS improved from 25.2 → 65.8	Arthroplasty after gunshot improved HHS; worse in contaminated cases
Özden et al*.* [3]	26	~ 47 (range 12–85)	Infection: 6/26 (23%)	HHS improved from ~ 53.0 → ~ 79.9	Significant improvement but high infection with shell fragments
Almirah et al*.* [9]	10	31.9 ± 11.8	2/10 (20%; SSI, HO)	HHS improved from 32.1 → 69.2	THA significantly improved function: complications associated with age and timing
Naziri et al*.* [6]	4	26 ± (12–48)	None reported	Clinically improved function (no formal score)	THA effective for post-traumatic arthritis after GSI
Sethi/Bartkiw et al*.* [7]	~ 7 procedures (fracture focus)	Not defined	Healing and low long-term instability	Non-arthroplasty procedures mainly described	Orthopaedic intervention for intra-articular ballot wounds indicated; THR described only in rare revisions
Bell et al*.* [12]	1	~ 12	None	Excellent outcome	Case report of staged THA after femoral neck GSI

Although direct comparison across studies is limited by heterogeneity in patient populations, injury patterns, and timing of intervention, the overall trend indicates that acceptable functional outcomes can be achieved with appropriate surgical strategy, even in complex and contaminated cases. Notably, our study demonstrated a clear inverse relationship between injury severity and functional outcome, a finding that has not been consistently emphasized in prior reports and may provide additional insight into prognostic stratification in this patient population. The mean HHS in our Group I patients (92 ± 5) approaches outcomes reported in conventional primary arthroplasty populations.

Across studies, key themes emerge staged or delayed THR improves functional outcomes; infection remains the primary complication, especially in contaminated wounds; fragment removal is critical to prevent secondary arthritis; and modern implant techniques enhance prosthetic stability [Table 3]. A recent systematic review by Tisnovsky et al. [4] examined 47 studies on gunshot-related hip injuries, highlighting variability in management strategies and the need for more prospective evidence to guide optimal treatment approaches. Our series builds upon these principles with a standardized, algorithmic approach, demonstrating low complication rates and favorable mid-term function in three clearly defined injury groups [1–12].

This study is limited by its retrospective, single-center, non-randomized design, heterogeneity in injury patterns and referral timing, and follow-up duration insufficient to assess long-term implant survival. Findings may therefore be less generalizable to centers with lower case volume or less specialized reconstructive expertise. The proposed framework does not represent a validated classification system or a prospective decision-making algorithm. Rather, it reflects an experience-based synthesis of injury patterns and corresponding reconstructive strategies observed within this cohort and is intended for descriptive and educational purposes rather than predictive stratification. Inter- and intraobserver reliability were not assessed, as the framework was not designed as a formal classification. Consequently, conclusions regarding optimization, reproducibility, or prognostic value should be interpreted with caution. Another limitation of this study is the absence of standardized patient-reported outcome measures (PROMs). While the Harris Hip Score (HHS) and WOMAC index provide valuable clinical assessment, they may be influenced by observer bias and may not fully capture the patient’s subjective experience. Another limitation is the absence of standardized functional photographs or video-based gait assessments at final follow-up. Because many patients were referred from conflict-affected regions outside our country and returned to their home locations following recovery, routine acquisition of standardized photographic or video documentation was not feasible. Functional outcomes were therefore assessed using validated clinical scoring systems (HHS and WOMAC), mobility status, and serial radiographic follow-up which represent the outcome measures most commonly reported in arthroplasty literature.

Future prospective studies incorporating validated PROMs would provide a more comprehensive evaluation of functional outcomes and multi-center studies are needed to evaluate external validity and reproducibility.

## Conclusion

Total hip replacement following gunshot hip injury is a complex but increasingly feasible reconstructive option. Optimal outcomes depend on careful patient selection, accurate assessment of contamination, thorough debridement, appropriate surgical timing, and meticulous reconstruction using modern implants, bone grafting, and dual-mobility constructs. Despite the challenges posed by extensive contamination or delayed presentation, acceptable mid-term outcomes can be achieved when reconstructive strategies are tailored to injury severity and contamination.

The severity-based framework described reflects institutional experience and highlights key injury characteristics influencing reconstructive planning. While not a validated classification or predictive tool, it provides pragmatic guidance for managing gunshot hip injuries and underscores the need for future external validation through larger, multi-center prospective studies.

## Data Availability

The datasets used and/or analyzed during the current study available from the corresponding author on reasonable request.
